# Characterization of Polydioxanone in Near-Field Electrospinning

**DOI:** 10.3390/polym12010001

**Published:** 2019-12-18

**Authors:** William E. King, Yvonne Gillespie, Keaton Gilbert, Gary L. Bowlin

**Affiliations:** 1 Department of Biomedical Engineering, University of Memphis, Memphis, TN 38152, USA; 2 Department of Biomedical Engineering, University of Tennessee Health Science Center, Memphis, TN 38163, USA

**Keywords:** near-field electrospinning, polydioxanone, fiber writing

## Abstract

Electrospinning is a popular method for creating random, non-woven fibrous templates for biomedical applications, and a subtype technique termed near-field electrospinning (NFES) was devised by reducing the air gap distance to millimeters. This decreased working distance paired with precise translational motion between the fiber source and collector allows for the direct writing of fibers. We demonstrate a near-field electrospinning device designed from a MakerFarm Prusa i3v three-dimensional (3D) printer to write polydioxanone (PDO) microfibers. PDO fiber diameters were characterized over the processing parameters: Air gap, polymer concentration, translational velocity, needle gauge, and applied voltage. Fiber crystallinity and individual fiber uniformity were evaluated for the polymer concentration and translational fiber deposition velocity. Fiber stacking was evaluated for the creation of 3D templates to guide the alignment of human gingival fibroblasts. The fiber diameters correlated positively with polymer concentration, applied voltage, and needle gauge; and inversely correlated with translational velocity and air gap distance. Individual fiber diameter variability decreases, and crystallinity increases with increasing translational fiber deposition velocity. These data resulted in the creation of tailored PDO 3D templates, which guided the alignment of primary human fibroblast cells. Together, these results suggest that NFES of PDO can be scaled to create precise geometries with tailored fiber diameters for biomedical applications.

## 1. Introduction

The modern exploration of electrospinning was credited to the Reneker group in the 1990s. [1,2]. This group formally investigated the process, processing conditions, fiber morphology, and suggested possible applications of electrospinning. Traditional solution electrospinning (TES) principally consist of a charged polymer solution and a counter electrode. The resulting electrostatic field exerts a force on the solution to form a Taylor cone [3,4]. If the force exceeds the surface tension of the solution, a liquid jet is extruded and accelerated towards the counter electrode. As the solvent evaporates in the air gap, a fiber is formed, while simultaneously incurring Rayleigh, axisymmetric, and bending instabilities [5]. These instabilities chaotically whip the fiber, which results in elongation and thinning, until it is randomly deposited on the collecting surface as a non-woven, fibrous material [2,6]. Researchers developing controlled fiber geometries and pore sizes have published numerous techniques, such as rotating and translating the collecting mandrel, the creation of precise electric fields, and the addition of porogens [7,8,9]. Each of these techniques confers a degree of control, but none allow for the individual placement of fibers to create custom fiber and pore geometries.

In 2003, Kameoka et al. demonstrated a method of controlling individual electro-spun fibers in a process that would later be termed near-field electrospinning (NFES). Specifically, they demonstrated a micro-fabricated silicone tip translating at an air gap distance of 5–15 mm over a counter electrode to write an individual fiber [10]. As with TES, this technique utilizes a charged polymer, in solution or melted with heat, to extrude a microfiber on a counter electrode, but the high degree of fiber control with this method arises from positioning the Taylor cone significantly closer to the counter electrode before any bending instabilities can occur. When paired with precise translational movement between the polymer source and collector, fibers can be directly “written” allowing for the creation of specific geometries that can be laid down layer-by-layer. As both NFES and TES utilize a Taylor cone formed in an electric field, it suggests that if a polymer solution/melt has the viscosity, conductivity, and surface tension of TES, then NFES is also possible [11]. To date, numerous polymers have successfully NFES, including but not limited to, polyethylene oxide (PEO), polyvinylpyrrolidone (PVP), polycaprolactone (PCL), polystyrene (PS), and polyvinylidene fluoride (PVDF) [12]. Early NFES setups used an “ink and quill” approach to write fibers onto a grounded substrate. However, NFES have progressed in using motion platforms or modified commercially available three-dimensional (3D) printers to position a spinneret with a continuous polymer source, therefore, allowing continuous fiber deposition with precise placement [10,11,13].

In this paper, we investigate the polymer polydioxanone (PDO) using a solution-based NFES apparatus custom designed from a commercial 3D printer. PDO has been used extensively in the medical field as a suture material termed “PDS II”. Its minimal inflammatory response, elastic mechanical properties, *in vivo* degradation rate of 6–8 weeks, and shape memory have resulted in researchers investigating TES microfibers of the material for other biomedical applications, such as drug delivery and tissue engineering [8,14,15,16,17,18,19]. To develop additional applications, we report PDO fiber diameter trends as a function of the major NFES processing parameters, including air gap distance, spinneret needle gauge, fiber deposition velocity, applied voltage, and polymer concentration. A subset of these parameters was further evaluated for their effects on fiber diameter variability and fiber crystallinity. Together, these results were used to create custom tailored 3D PDO templates, which were evaluated in a preliminary study for contact guidance induced alignment of human gingival fibroblast. This preliminary work demonstrates that PDO can be near-field electro-spun to create templates with tailorable characteristics that may be advantageous for biomedical applications.

## 2. Materials and Methods

### 2.1. NFES Apparatus

**Figure 1:**
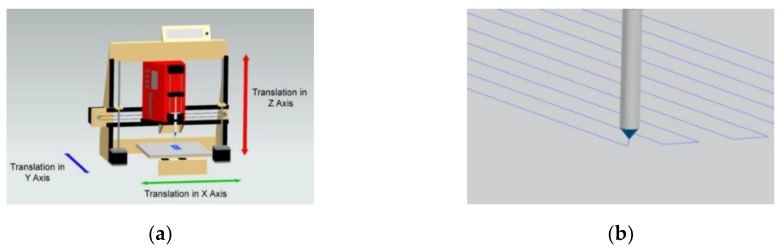
(**a**) Schematic of a custom modified three-dimensional (3D) printer used to create the NFES apparatus. (**b**) Representative depiction of precisely written NFES fibers.

A consumer 3D printer (Prusa 8″ i3v Kit V-Slot Extrusion, Makerfarm, South Jordan, UT, USA) was modified by removing the filament extrusion print head to accommodate the NFES print head, as inspired from the work of Fattahi et al. (Figure 1a,b) [13]. The NFES print head consisted of a NE-300 Just Infusion^TM^ syringe pump (New Era Pump Systems, Inc., Farmingdale, NY, USA) fixed on a custom designed mounting base, polypropylene syringe, and a blunt luer-lock needle charged by a DC voltage source (HV050REG(-), Information Unlimited, Amherst, NH, USA). The print head was able to translate in the *X*- and *Z*-axis, while the grounded collector translated in the *Y*-axis. Translational path and velocity were written in g-code and relayed to the 3D printer using the software Repetier (Hot-World GmbH and Co. KG, Willich, Germany).

### 2.2. Fiber Diameter Characterization

#### 2.2.1. Setup and Materials

Polydioxanone (DIOXOMAXX^®^ 100, Inherent viscosity 2.13 dL/g, Bezwada Biomedical, LLC, Hillsborough, NJ, USA) solutions were dissolved overnight in 1,1,1,3,3,3-hexafluoro-2-propanol (HFP, Oakwood Products, Inc., Estill, SC, USA). Fibers were deposited on glass cover slides (Cat No. 2980-246, 24 × 60 × 1.5 mm, Corning, Inc., Corning, NY, USA) for rapid visualization. The slides were prepared with corona plasma treatment (Model: BD-20, Electro-Technic Products, Chicago, IL, USA) at the time of use to facilitate the NFES process on an insulator [13]. The plasma treatment spring tip (Model: 12,201 Spring Tip, Electro-Technic Products, Chicago, IL, USA) was positioned perpendicularly, 2 mm above the glass cover slide, and passed over the slide at a rate of 2 passes per second for 60 s. Treated slides were affixed to a 6″ × 6″ precision ground, electrically grounded aluminum plate (3511T151, McMaster-Carr, Elmhurst, IL, USA) on the NFES print bed. A voltage of −1.8 kV was applied for air gap distances less than 2 mm and −2.0 kV for distances greater than or equal to 2 mm to rapidly initiate fiber formation in under 10 s. This measure was implemented as excess solvent evaporation introduces variability and can inhibit fiber formation in the NFES process. After initial fiber extrusion, the applied voltage was immediately reduced to a target voltage, and the NFES print head translated at 10 mm/s for 60 s to allow for fiber deposition stabilization.

#### 2.2.2. Fiber Diameter Processing Parameters

Fiber deposition was evaluated over the systematically varied processing parameter air gap, polymer concentration, needle gauge, translational velocity, and applied voltage for their effect on fiber diameter (Table 1). Independent parameters were chosen to provide continuous, robust fiber deposition throughout the range of dependent parameters. When evaluating needle gauge as the dependent variable, constant polymer velocity was used in order to evaluate the effect on fiber diameter without polymer volumetric flow confounding the results. For a set of parameters, each replicate (*n* = 5) was comprised of 60 parallel fibers that were 40 mm in length and a fiber center to center spacing of 0.15 mm.

#### 2.2.3. Fiber Variability Processing Parameters

**Figure 2:**
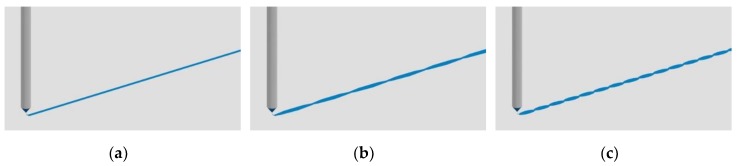
Graphical depiction of a NFES fiber’s variability in diameter along its length for (**a**) no variability; (**b**) mild variability; (**c**) extreme variability.

Polymer concentration and translational velocity were systematically varied to measure their effect on fiber diameter variability (Table 2). Variability in this context was defined as the extent of diameter undulating along the length of an individual fiber (Figure 2a–c). This characteristic is important as sharply transitioning geometries, such as “beading,” in electro-spun fibers can result in poor cellular adhesion and proliferation [20]. Independent parameters were chosen to provide continuous, robust fiber deposition throughout the range of dependent parameters. Replicates (*n* = 3) consisted of a 40 mm long fiber randomly sampled from an array of 60 fibers.

#### 2.2.4. Fiber Imaging and Diameter Analysis

Fibers on glass coverslips were imaged with a digital camera microscope at 500× (VHX-1000 or 6000, Keyence, Itasca, IL, USA). Greyscale images were converted to black and white using a threshold filter in Photoshop CC 2017 (Adobe, Inc., San Jose, CA, USA). Fiber diameters were measured from acquired images using Fibraquant v1.3.149 (nanoScaffold Technologies, Chapel Hill, NC, USA). The software was calibrated using the number of pixels comprising the length of each image scale bar to convert pixels to length. Fiber means and standard deviations were determined from a minimum of 60 semi-automated random measurements per image. Fiber for processing parameter characterization were imaged in three random locations. Individual fibers for variability analysis were imaged in four random locations.

### 2.3. 3D NFES Templates

#### 2.3.1. Setup and Materials

Individual fibers were sequentially stacked to create uniaxial ribbon templates. The *Y*-axis print bed was translated 40 mm for 80 cycles using a 26 gauge, 2″ blunt tip needle. PDO solutions of 200 mg/mL were dispensed with a flow rate of 15 μL/h, an applied voltage of −1.4 kV, and with a starting *Z*-axis air gap of 1.8 mm. The NFES print head translated in the *Z*-axis 0.1 mm every 20 cycles to account for the changing height. Biaxial grid templates were created by depositing parallel fiber layers with a 90° offset. Four, twelve, and thirty parallel fiber layers were written with the NFES print head translating 14.4 mm in the *X*-axis and the NFES print bed, translating 40 mm in the *Y*-axis. The four layers of stacked fibers were programmed with a spacing of 0.2 mm in the *X*-axis and 0.15 mm in the *Y*-axis. Both twelve and thirty stacked fiber layers were programmed with a spacing of 0.1 mm the *X*-axis and 0.075 mm in the *Y*-axis. The resulting templates were placed in a vacuum chamber (Isotemp^®^ Vacuum Oven Model 281A, Fisher Scientific, Rockingham County, NH, USA) for 10 min at a minimum of 70 kPa below atmospheric pressure and at a temperature of 23 °C to degas any excess solvent.

#### 2.3.2. Template Imaging and Fiber Diameter Analysis

A small sample was dissected from the stacked fiber templates for imaging. Templates were sputter coated in an argon gas field with 5.0 nm of 60:40 gold-palladium. Samples were visualized using a scanning electron microscope (Nova Nano 650 FEG, FEI Co., Hillsboro, OR, USA) with the field emission gun set to +20 kV, a spot size of 3 and 5 mm working distance. Fiber diameters were measured from acquired images using Fibraquant, as described in Section 2.2.4.

### 2.4. Fiber Crystalinity

#### 2.4.1. Fiber Processing Parameters

Polymer concentration and translational velocity were systematically varied to measure their effect on resulting fiber crystallinity (Table 3). Parallel fiber layers with a 90° offset were stacked to create biaxial grid templates for analysis as described in Section 2.3.1 with a fiber spacing of 0.1 mm in the *X*-axis and 0.075 mm in the *Y*-axis. Independent parameters were chosen to provide continuous, robust fiber deposition throughout the range of dependent parameters. As a comparison, TES samples were created with a PDO concentration of 140 mg/mL at +25 kV, 17.8 cm air gap, 4 mL/h, through an 18 gauge, 2″ length needle to create fiber diameters on the same order of magnitude as NFES. TES fibers were collected on a grounded, stainless steel mandrel (rectangular dimensions: 200 × 750 × 5 mm^3^) rotating at 1250 rpm, and translating 6.5 cm/s over a distance of 13 cm. The fibers were imaged and diameters analyzed as described in Section 2.3.2.

#### 2.4.2. Differential Scanning Calorimetry (DSC)

For each sample, melt enthalpy was measured using a calorimeter (200 PC DSC, Netzsch, Burlington, MA, USA). The sample mass ranged from 4–5 mg and was placed in aluminum weigh tins (DSC72006, DSC Consumables, Inc., Austin, MN, USA). Tinned samples and an empty reference tin were heated at 10 °C/min from 25 to 140 °C [21]. Melt enthalpy was calculated through the integration of the melting curve using Proteus v4.3.1 9 (Netzsch, Burlington, MA, USA). PDO fiber crystallinity was experimentally determined by comparing the stock PDO melt enthalpy of known crystallinity with the experimental samples [22]. Theoretical melt enthalpy for a 100% crystalline PDO polymer was calculated from the measured melt enthalpy of an average 42.5% crystalline stock PDO (Table 4). Sample melt enthalpy was compared to the theoretical 100% crystalline melt enthalpy to ascertain sample crystallinity.

### 2.5. Template-Driven Cell Alignment

#### 2.5.1. Template Preparation Processing Parameters

Biaxial grid templates were created, as described in Section 2.3.1, using the processing parameters 140 mg/mL PDO, 15 μL/h flow rate, 30 mm/s translational velocity, 1.8 mm air gap, and −1.3 kV applied voltage, through a 2″, 23-gauge needle. Templates had a fiber spacing of 0.1 mm in the *X*-axis and 0.075 mm in the *Y*-axis. TES templates were generated from PDO solutions of 160 mg/mL, a flow rate of 0.8 mL/h, an applied +8 kV voltage, and an air gap of 15.2 cm through a 14 gauge, 2″ needle. Fibers were collected on a mandrel described in Section 2.4.1. PDO film of 0.16 mm thickness was created from a 100 mg/mL cast solution. These templates and films were imaged and fiber diameters analyzed as described in Section 2.3.2. Samples 6 mm in diameter were created with a medical biopsy punch (Acu-Punch, 6.0 mm, Acuderm Inc., Fort Lauderdale, FL, USA). Tissue culture plastic disks (Petri Dish, Stackable Lid, 60 × 15 mm^2^, Fisher Scientific, Rockingham County, NH, USA) 6 mm in diameter were cut out using a laser CNC (LS100ex, Gravograph, Auvergne-Rhône-Alpes, France) to serve as a control. Samples were UV sterilized (EN-280L 1090 µW/cm^2^ at 15 cm, Spectroline^®^, Westbury, NY, USA) for 10 min at a distance of 10 cm from the source on each side, placed in a 96-well plate, and secured with 4 mm inner diameter glass cloning cylinders.

#### 2.5.2. In Vitro Cell Culture

Primary human gingival fibroblasts (HGF-1, CRL-2014, ATCC, Manassas, VA, USA) were cultured in Dulbecco’s Modified Eagle Medium/Nutrient Mixture DMEM/F-12 (HyClone^TM^, GE Health Life Sciences, South Logan, UT, USA) with 10% fetal bovine serum (S11150, Atlanta Biologicals, Flowery Branch, GA, USA) 100 IU Penicillin (Corning, Inc., Corning, NY, USA), 100 μg/mL Streptomycin (Corning, Inc., Corning, NY, USA) and 2 mM L-glutamine (GlutaMAX^TM^, Life Technologies, Carlsbad, CA, USA) All cells used in the experiments were at passage 7. Replicates (*n* = 3) were seeded at a concentration of 50,000 cells/well and incubated for 3 days at 37 °C and 5% CO_2_. Cells were fixed with 3.7% paraformaldehyde for 30 min, permeabilized with 0.1% Triton X-100 for 10 min at room temperature, washed with phosphate buffered saline (PBS) 3 times, and stained with Actin green and 4′,6-diamidino-2-phenylindole (DAPI) per manufacture instructions (ActinGreen^TM^ 488 ReadyProbes^TM^, Invitrogen, Carlsbad, CA, USA; NucBlue^TM^ Fixed Cell Stain ReadyProbes^TM^, Molecular Probes, Eugene, OR, USA). Templates were stored in 96 well plates immersed in PBS at 4 °C and were imaged within 72 h.

#### 2.5.3. Fluorescent Microscopy and Analysis

Templates were mounted using PBS on glass slides with a coverslip for imaging. Templates were imaged for quantification in three representative locations with an Olympus BX 43 fluorescent microscope (Olympus Corporation, Tokyo, Japan) at 20× magnification. Actin green stained cells were analyzed for their orientation using the program CellProfiler^TM^ v3.1.8 (Broad Institute, Cambridge, MA, USA) [13,23]. Specifically, imaged pixels of cells were isolated as objects and their orientation quantified relative to the *X*-axis.

### 2.6. Statistics

All statistical analysis was performed in Prism 8 v8.2.1 (GraphPad Software Inc., San Diego, CA, USA). Differences for fiber diameter, variability, and crystallization were tested using ANOVA with Dunnett multiple comparisons at a significance of *p* < 0.05. Trends in the data were tested using the Pearson correlation coefficient at a significance of *p* < 0.05. Comparing the means of the cell alignment data was not meaningful due to the symmetric data about zero. Differences in the cumulative distribution of these data were tested using the Kolmogorov-Smirnov test with Bonferroni multiple comparisons correction at a corrected significance of *p* < 0.05.

## 3. Results

### 3.1. PDO Fiber Characterization

#### 3.1.1. Processing Parameters

**Figure 3:**
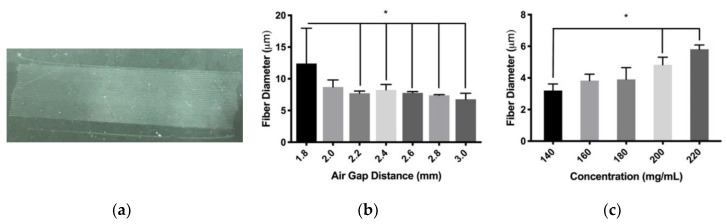

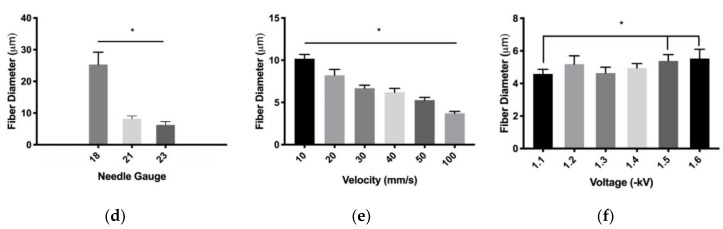
NFES PDO fiber characterization (**a**) Representative photo of 60 parallel fibers on a glass substrate. Fiber diameter response to (**b**) air gap (* indicates *p* < 0.05 compared to 1.8 mm), (**c**) polymer concentration (* indicates *p* < 0.05 compared to 140 mg/mL), (**d**) needle gauge (* indicates *p* < 0.05 compared to 18 gauge), (**e**) translational velocity (* indicates *p* < 0.05 compared to 10 mm/s), and (**f**) Applied voltage (* indicates *p* < 0.05 compared to −1.1 kV). Data are presented as mean ± standard deviation.

The NFES setup demonstrated the creation of orderly PDO fibers for fiber diameter characterization over a range of processing parameters (Figure 3a). The processing parameter of air gap distance showed that the fiber diameter decreased from 12.4 ± 5.6 to 6.8 ± 0.9 μm over a range of 1.2 mm (Figure 3b). The differences were detected between the 1.8 mm parameter and all other air gap parameters with the exception of 2.0 mm. The relationship between air gap distance and fiber diameter had a significant Pearson correlation score of r = −0.53. These results suggest that fiber diameter is inversely proportional to the distance between the needle tip and the collector. A change in PDO concentration from 140 to 220 mg/mL demonstrated an increase in fiber diameter from 3.2 ± 0.4 to 5.8 ± 0.3 μm (Figure 3c). Differences were detected between 140 mg/mL compared to the 200 and 220 mg/mL parameters, and the relationship between polymer concentration and fiber diameter had a significant Pearson correlation score of r = 0.87. Therefore, these data suggest that fiber diameter positively correlates with polymer concentration. A decrease in the inner diameter of the polymer dispensing needles from 18 to 23 gauge showed a decrease in the average fiber diameter from 25.3 ± 3.9 to 6.3 ± 1.1 μm (Figure 3d). Differences were detected between the 18 and 21 gauge needles as well as 18 and 23 gauge needles. Furthermore, the relationship between the needle diameter and fiber diameter had a significant Pearson correlation score of r = −0.92. Together, these data suggest an inverse correlation with fiber diameter and the polymer dispensing needle gauge. An increase in the relative translational velocity from 10 to 100 mm/s between the polymer dispensing needle and collecting substrate showed a decrease in the average fiber diameter of 10.2 ± 0.5 to 3.7 ± 0.2 μm (Figure 3e). Differences were observed between all parameters compared to 10 mm/s, and the relationship between relative translational velocity and fiber diameter had a significant Pearson correlation score of r = −0.89. These data suggest that fiber diameter is inversely proportional to the relative translational velocity. An increase in the applied voltage from −1.1 to −1.6 kV showed an increase in the average fiber diameter from 4.6 ± 0.3 to 5.5 ± 0.6 μm (Figure 3f). Differences were observed between the −1.1 and −1.5 kV parameters as well as −1.1 and −1.6 kV parameters. In addition, the relationship between voltage and fiber diameter had a significant Pearson correlation score of r = 0.54. Together, these data suggest that fiber diameter is proportional to the applied voltage. The totality of these data suggests that NFES PDO fiber diameters are tailorable over the range of every major processing parameter.

#### 3.1.2. Fiber Variability

**Figure 4:**
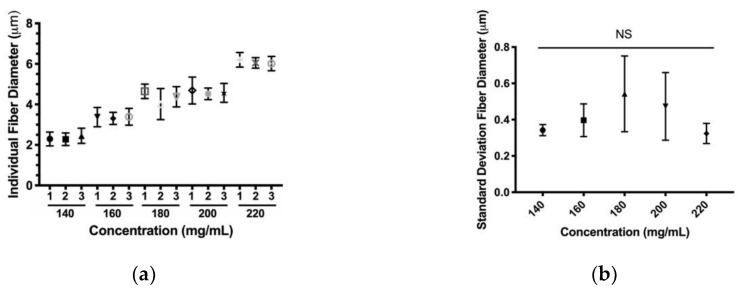
Individual NFES PDO fibers of increasing polymer concentration (**a**) Fiber diameter, (**b**) Standard deviation. Data are presented as mean ± standard deviation.

**Figure 5:**
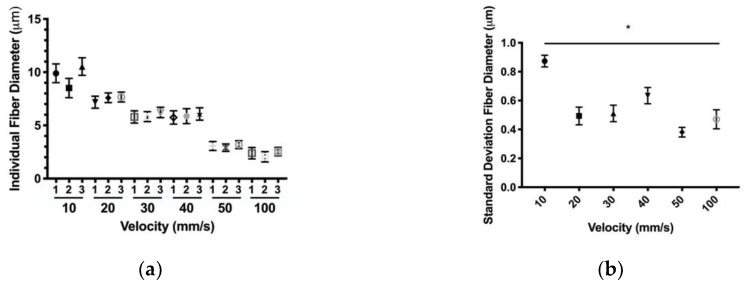
Individual NFES PDO fibers of increasing translational velocity (**a**) Fiber diameter, (**b**) Standard deviation (* indicates *p* < 0.05 compared to 10 mm/s). Data are presented as mean ± standard deviation.

Translational velocity and polymer concentration were chosen as parameters of interest to examine, as they were the most robust parameters for producing smaller fiber diameters. Both parameters’ effects on fiber diameter variability along the length of an individual fiber were evaluated. These data for individual fibers further support the trends found in the previous section, (Figure 4a and Figure 5a). For a given polymer concentration parameter, the average diameters of each fiber were all within one standard deviation of each other. Standard deviations of the fibers were plotted to ascertain trends in variability (Figure 4b and Figure 5b). Over the PDO polymer concentration range, there was no significance between the 140 mg/mL parameter and any other parameter. Furthermore, the data showed no correlation with a non-significant Pearson correlation score of 0.01 (*p* > 0.05). For a given translational velocity parameter, the average diameters of each fiber were all within one standard deviation of each other with the exception of the 10 mm/s parameter. Over the translational velocity range, there was a significance between the 10 mm/s parameter and any other parameters. These data also showed a significant Pearson correlation score of r = −0.51. Together, these results suggest PDO solution concentration does not affect individual fiber diameter variability, but variability in diameter is inversely proportional to translational velocity.

#### 3.1.3. Fiber Stacking

**Figure 6:**
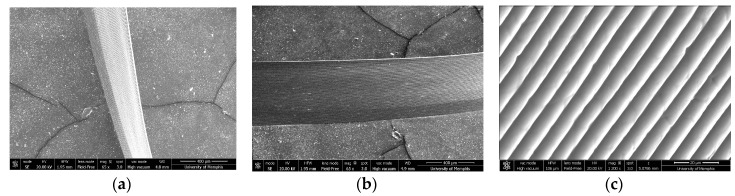
(**a**) Top down view of 80 stacked PDO fibers (Scale bar = 400 μm). (**b**) Alternative view of stacked fiber at a 40° tilt. (Scale bar = 400 μm). (**c**) Magnified view of stacked fibers at a 40° tilt (Scale bar = 20 μm).

**Figure 7:**
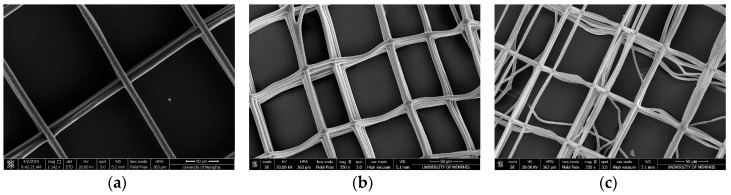
Biaxial grid stack layers of PDO fibers (**a**) Four layers. (**b**) Twelve layers (**c**) Thirty layers. (Scale bars = 50 μm).

Three-dimensional NFES constructs were created though stacking layers of fibers. Uniaxial ribbon fiber stacking was first evaluated (Figure 6a–c). Scanning electron micrograph (SEM) imaging show uniform stacking of all 80 PDO fibers from the programmed 80 cycles of fiber deposition. These data suggest that a systematic layering of PDO fibers is possible. Subsequently, the systematic layering of biaxial grid-oriented fibers was evaluated. Parallel fibers were written with each layer defined by a 90° rotational offset. Four, twelve, and thirty layers of fibers were stacked (Figure 7a–c). SEM imaging demonstrated that PDO fibers could be bi-axially stacked into grids. In addition, these data suggest that, an increasing number of layers results in a corresponding increased number of qualitative errors in fiber deposition, as evidenced by the few disorderly fibers.

### 3.2. Fiber Crystalinity

**Figure 8:**
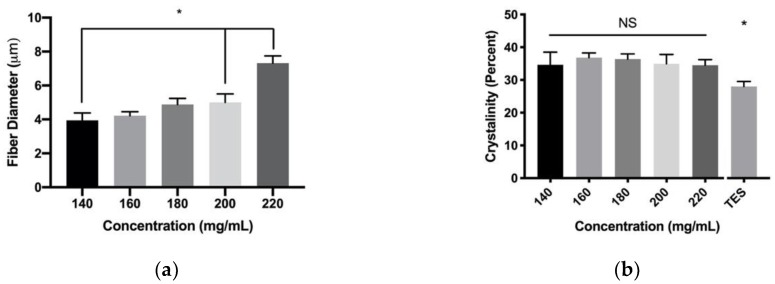
Varied PDO concentration on whole template (**a**) Fiber diameter (* indicates *p* < 0.05 compared to 140 mg/mL) and (**b**) Percent crystallinity of NFES fibers and TES fibers (* indicates *p* < 0.05 compared to all NFES groups). Data are presented as mean ± standard deviation.

**Figure 9:**
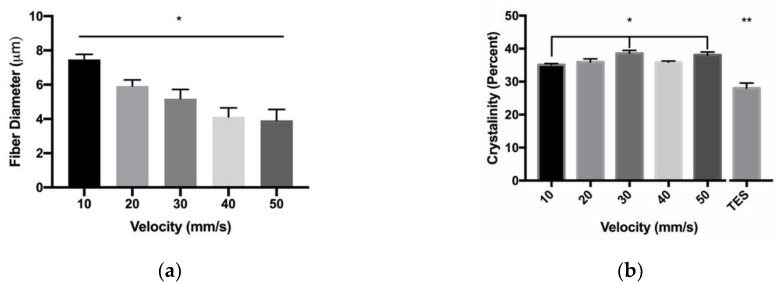
Varied translational velocity on whole template (**a**) Fiber diameter (* indicates *p* < 0.05 compared to 10 mm/s); and (**b**) Percent crystallinity of NFES fibers and TES fibers (* indicates significantly different compared to 10 mm/s, ** indicates significantly different to all NFES groups). Data are presented as mean ± standard deviation.

Translational velocity and PDO concentration were further evaluated for their effects on fiber crystallinity. Over the evaluated range of PDO concentrations, the biaxial grid templates demonstrated the same fiber diameter trends, that were observed with a single layer of fibers (Figure 8a). The differences in fiber diameters were detected between 140 mg/mL, compared to the 200 and 220 mg/mL parameters. There was no significant difference detected in fiber crystallinity in 140 mg/mL, compared to all other concentrations (Figure 8b). When compared to the TES constructs made from 140 mg/mL PDO, there was a significant average increase of 7.4% crystallinity, compared to all NFES parameters. Over the evaluated range of translational velocity, the biaxial grid templates demonstrated the same fiber diameter trends observed with a single layer of fibers (Figure 9a). The differences in fiber diameters were detected between all parameters and 10 mm/s. There was a significant difference in fiber crystallinity in the 10 mm/s parameter at 35.1 ± 0.3% crystalline compared to 30 as well as the 50 mm/s parameter at 38.6 ± 0.9% and 38.0 ± 0.9% crystalline, respectively (Figure 9b). Furthermore, these data demonstrated a significant Pearson correlation score of r = 0.56. When compared to the 140 mg/mL PDO TES constructs, these data showed a significant average increase of 8.7% in crystallinity compared to all NFES parameters. Together, these data suggest that based on these methods, we were unable to detect an effect of PDO polymer concentration on fiber crystallinity. There is evidence to suggest a positive correlation between translational velocity and fiber crystallinity. All NFES processing parameters evaluated showed a significant increase in fiber crystallinity when compared to TES constructs of 140 mg/mL PDO, suggesting the shorter air gap distance of NFES results in increased fiber crystallinity.

### 3.3. In Vitro Cell Culture

**Figure 10:**
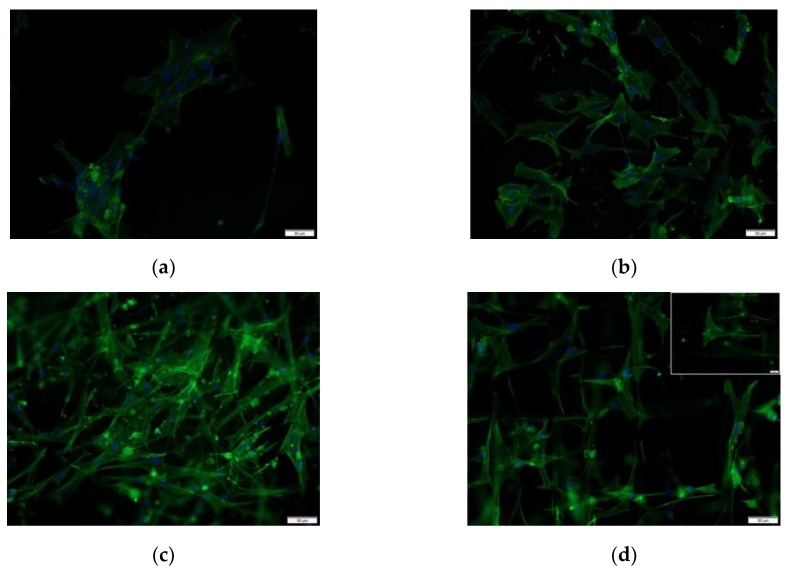
DAPI and Actin Green stained human gingival fibroblasts at 20× magnification (Scale bar = 50 μm) on (**a**) TCP; (**b**) PDO film; (**c**) TES; and (**d**) NFES (representative inset displaying only actin green channel at 40× magnification, Scale bar = 20 μm).

**Figure 11:**
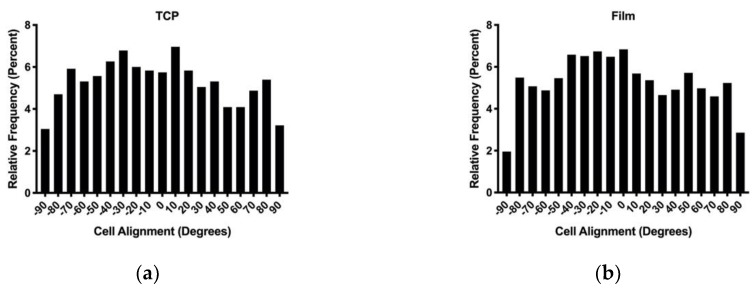

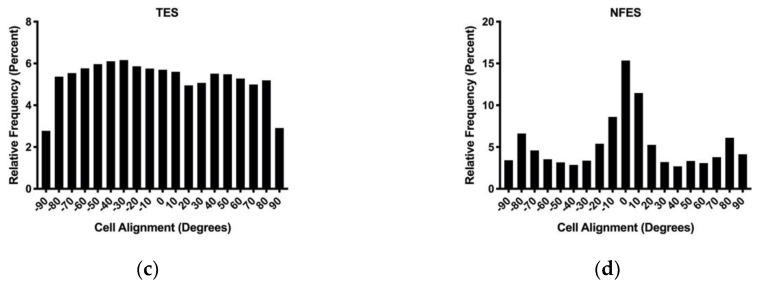
Histogram of percent cell alignment between −90 and 90° for (**a**) TCP; (**b**) PDO membrane; (**c**) TES constructs; (**d**) NFES constructs.

In order to evaluate the effects of contact guidance induced alignment, human gingival fibroblasts were seeded onto TCP, PDO films, TES templates, and NFES templates (Figure 10a,d). The actin cytoskeleton was quantified for its orientation relative to the *X*-axis (Figure 11a,d). These data show that human fibroblast cells are randomly distributed on TCP, PDO film, and TES templates. In contrast, biaxial grid NFES templates demonstrate a sharp peak at 0° with 57% of the total area from ± 45° and minor peaks at ± 80°–90° with a combined area of 43% from ± 45°–90°, indicating 90° biaxial distribution. Additionally, the cumulative frequency distribution of NFES compared to the TCP, membrane, and TES substrates were each found to be significantly different, while there was no significant difference among the non-NFES substrates. These data support that oriented NFES fibers can be used as a template to guide cell alignment.

## 4. Discussion

In this work, we demonstrated the novel creation of near-field electro-spun PDO microfibers. A low cost, commercially available 3D printer was modified with a custom mounted syringe pump and counter electrode for NFES functionality. Our results suggest that PDO is a reliable biodegradable polymer for the creation of NFES written fibers. The trends reported in our characterization of the major NFES processing parameters for PDO align with other reported NFES polymers [24,25,26,27,28,29,30]. These parameter trends are also consistent with TES parameter trends, except for the applied voltage. Along with others in the literature, we reported that fiber diameter is proportional to the applied voltage, while an inversely proportional trend is observed in TES [31,32]. Applied voltage producing an electrostatic field is the singular driving force of both NFES and TES. The mechanism for the opposing observed trends is thought to be the air gap distance. For TES, the electrostatic field also acts on the charges on the fiber in the air gap, resulting in greater repulsions. These repulsions serve to whip and elongate the fiber resulting in a reduction of the fiber diameter. Without this air gap, an increased driving force further accelerates the fiber without bending instabilities, leading to an increase in fiber diameter.

Over the observed range of air gap distances, the 1.8 mm gap had a standard deviation approximately nine times greater than the other distances. We hypothesized that, for a given set of parameters, a reduction of the air gap results in an increased accelerating force on the polymer. At a critical distance, the polymer solution is accelerated toward the grounded collector such that the cohesion of the viscous solution pulls along additional solution. Air gaps below the critical distance will result in complete depletion of the Taylor cone, while greater distances exist in a mass transfer equilibrium. The resultant reduction of polymer in the Taylor cone temporarily increase the effective air gap distance between the tip of the Taylor cone and the collector. This distance is now above the critical distance and results in a reduction of polymer accelerated to the collector. As the polymer is constantly supplied via a syringe pump, the Taylor cone receives a net gain of polymer, until the critical effective air gap distance is achieved. This system continues to cycle through this process resulting in a larger variation of fiber diameters. The air gap parameter had the weakest correlation of all the processing parameters. Based on our hypothesis if the 1.8 mm gap was excluded, the Pearson correlation score would result in a stronger correlation from r = −0.53 to r = −0.61.

Translational velocity and PDO polymer concentration processing parameters were chosen to evaluate further as they were the most robust for producing smaller fiber diameters. Fiber diameter variability along the length of individual fibers was evaluated over the range of these processing parameters. From these two parameters, we demonstrated that the increasing translational velocity decreases the fiber diameter variability along individual fibers. Translational velocity is the only processing parameter besides applied voltage, which exerts a force on the fiber. When the relative translational velocity exceeds the rate of fiber extrusion, a tensile force is exerted on the fiber. We hypothesize that this force is responsible for attenuating the variability observed along the length of the fiber. The characterization of the NFES PDO processing parameters and variability will allow for the creation of custom programmed constructs with tailored fiber diameters and tolerance, which is not possible with TES.

The vast majority of biomedical applications for microfibers are dependent on the creation of macro-sized 3D constructs. We briefly explored the extent of uni-axial ribbons and bi-axial grid fiber stacking of PDO with our NFES apparatus. We showed that fiber stacking was possible and that these constructs were easily manipulated for SEM imaging. As the number of layers increased, the imaging showed that the number of imperfections in the constructs also increased. Precise fiber stacking is a function of the precision of the 3D printer’s stepper motors, degree of wetness of the drying polymer fibers, residual charge repulsion effects, and system disturbance, such as vibration or ventilation air currents. In the current system, the translation of the syringe pump in the *X*-axis introduced vibrations at higher translational velocities. It is important to note that perfect fiber placement may not be necessary depending on the application.

In relation to the tailored fibers, the degree of fiber crystallinity has implications on mechanical property and degradation rates. PDO, as well as other polyesters, such as poly(lactide-co-glycolide) (PLGA) and PCL degrade hydrolytically under physiological *in vitro* conditions of PBS and 37 °C, as well as in vivo. Degradation specifically occurs in two stages: (1) Attack of the more solvent accessible amorphous regions followed by (2) attack of the more solvent resilient crystalline regions [33,34]. Therefore, there is a positive correlation with NFES parameters that increase fiber crystallinity and prolong degradation rates.

We were unable to detect a difference in fiber crystallinity with increasing PDO polymer concentration, but we were able to measure a small effect with increasing translational velocity. Between the effect observed and the translational velocity fiber characterization trend, we hypothesize that the tensile force exerted from increasing translational velocities mechanically draws out and elongates the fiber. This process results in fiber thinning and an increase in crystallinity. Both studied parameters demonstrate an increase in fiber crystallinity compared to the measured TES sample. Characterization of TES processing parameters on fiber crystallinity to fully elucidate the difference in NFES compared to TES fiber crystallinity was beyond the scope of this paper. Our preliminary data does suggest a possible systematic increase in fiber crystallinity, thereby, adding another degree of fiber tailorability.

Degradation rates were also found to be affected by fiber morphology [35,36]. The average fiber diameter has the multifactorial effect of total mass, surface area to volume ratio (SAVR), and crystallinity. Our results showed that increasing polymer concentration did not increase crystallinity, but did increase the average fiber diameter. This increase in fiber diameter increases the total mass to be degraded for a given length of fiber, as well as reduce the SAVR of the fiber for solvent-mediated hydrolysis at the fiber surface. Alternatively, increasing translational velocity increased the crystallinity, decreased fiber diameter, and increased SAVR. These results provide a basis for future work to study both in vitro and in vivo NFES PDO degradation, as well as to determine which factors dominate the effective degradation rate based on the specifications for individual applications.

Electrospun fibers are commonly used for biomedical and tissue engineering applications as adherent cells interact and align with the ECM-like features of these substrates. The absence of surface features for cells to adhere to in the TCP and PDO film samples were thought to result in a random distribution of cell orientation. Similarly, it was anticipated that the random distribution of fibers in the TES templates would also result in a random cellular orientation distribution. NFES constructs consisted of fibers oriented with 90° offsets, and therefore would be expected to show prominent peaks of actin alignment having similar 90° offsets. Our results demonstrate that NFES of PDO can act as a template to induce the alignment of human fibroblast cells to custom orientations and create a basis for future work exploring biocompatibility in vitro, with application-specific testing, followed by evaluating in vivo tissue integration and resolution.

NFES allows for the creation of highly precise fibers with custom programmed geometries, but this method is not without limitations. NFES has limited throughput, as flow rates are typically on the order of microliters per hour as opposed to TES with typical flow rates of milliliters per hour. Through the modification of a cost-effective 3D printer, we hope to ameliorate this issue with the scalability of multiple NFES units. Another current shortcoming of NFES is the lower limit of fiber diameter as the shortened air gap subsequently reduces the time of flight for fibers to thin and elongate. TES fiber diameters range from 200 nm to 2 μm, while NFES fibers range from 0.5–40 μm [37]. Human ECM ranges between 50–500 nm, and to date, NFES has been unable to create fibers in this range. Alternatively, there is evidence to suggest a favorable cellular response to fibers in the 1–2 μm range [38]. Ultimately, we have demonstrated that NFES is a powerful technology that enables the precise deposition of fibers to create unique biomaterials made of PDO, and with further optimization, NFES may enable the creation of unique biomaterials with previously unobtainable tailorability.

## 5. Conclusions

The NFES setup was demonstrated to allow for the creation of precise PDO microfibers. These fibers can be custom tailored in orientation, diameter, crystallinity, and layers for three dimensions of controlled fiber spacing and porosity. More importantly, we anticipate that this polymer and platform can be scaled up to create templates with complex geometries for biomedical applications. The most prominent of these applications is the creation of extracellular matrix-like environments with specific geometries to act as a biodegradable template and dictate cellular responses to guide in situ regeneration of tissues.

## Figures and Tables

**Table 1:** Systematically varied near-field electrospinning (NFES) processing parameters for polydioxanone (PDO) fibers.

Dependent Parameter	Range, (Increment)	Independent Parameters	Value
Air Gap	1.8–3.0 mm, (0.2)	Polymer Concentration:	180 mg/mL
		Needle Gauge:	23 gauge, 2″ length
		Translational Velocity:	30 mm/s
		Applied Voltage:	−1.3 kV
		Flow Rate:	15 μL/h
	
Polymer Concentration	140–220 mg/mL, (20)	Air Gap:	2.5 mm
		Needle Gauge:	23 gauge, 2″ length
		Translational Velocity:	30 mm/s
		Applied Voltage:	−1.2 kV
		Flow Rate:	15 μL/h
Needle Gauge	18,21, and 23 gauge, 2″ length	Air Gap:	1.8 mm
		Polymer Concentration:	180 mg/mL
		Translational Velocity:	30 mm/s
		Applied Voltage:	−1.2 kV
		Flow Rate:	23 gauge: 15 μL/h ^1^
			21 gauge: 33.7 μL/h ^1^
			18 gauge: 91.6 μL/h ^1^
Translational Velocity	10–50 mm/s, (10) And 100 mm/s	Air Gap:	1.8 mm
		Polymer Concentration:	180 mg/mL
		Needle Gauge:	23 gauge, 2″ length
		Applied Voltage:	−1.2 kV
		Flow Rate:	10 μL/h
Applied Voltage	1.1–1.6 kV, (0.1)	Air Gap:	1.8 mm
		Polymer Concentration:	160 mg/mL
		Needle Gauge:	23 gauge, 2″ length
		Translational Velocity:	30 mm/s
		Flow Rate:	15 μL/h

^1^. Equivalent to a constant polymer flow velocity of 0.0459 mm/s.

**Table 2:** NFES PDO fiber diameter variability parameters.

Dependent Parameter	Range (Increment)	Independent Parameters	Value
Polymer Concentration	140–220 mg/mL, (20)	Air Gap:	2.5 mm
		Needle Gauge:	23 gauge, 2″ length
		Translational Velocity:	30 mm/s
		Applied Voltage:	−1.1 kV
		Flow Rate:	15 μL/h
Translational Velocity	10–50 mm/s, (10) and 100 mm/s	Air Gap:	1.8 mm
		Polymer Concentration:	180 mg/mL
		Needle Gauge:	23 gauge, 2″ length
		Applied Voltage:	−1.1 kV
		Flow Rate:	10 μL/h

**Table 3:** NFES parameters for fiber crystallinity analysis.

Dependent Parameter	Range and Increment	Independent Parameters	Value
Polymer Concentration	140–220 mg/mL, (20)	Air Gap:	1.8 mm
		Needle Gauge:	23 gauge, 2″ length
		Translational Velocity:	30 mm/s
		Applied Voltage:	−1.3 kV
		Flow Rate:	15 μL/h
Translational Velocity	10–50 mm/s, (10)	Air Gap:	1.8 mm
		Polymer Concentration:	180 mg/mL
		Needle Gauge:	23 gauge, 2″ length
		Applied Voltage:	−1.2 kV
		Flow Rate:	15 μL/h

**Table 4:** Melt enthalpy and crystallization of stock PDO and representative calculated sample.

Sample	Melt Enthalpy (J/g)	Crystallinity	Theoretical Melt Enthalpy for 100% Crystallization (J/g)
Stock PDO	84.2	42.5%	198.5
140 mg/mL PDO	68.7	34.6%	−
